# *FOXO3* and *PTEN* expression in the ovary of girls with extra-gonadal cancer with or without chemotherapy treatment prior to cryopreservation

**DOI:** 10.1186/s12905-023-02648-x

**Published:** 2023-09-22

**Authors:** María Itatí Albamonte, Lara Y. Calabró, Mirta S. Albamonte, Alfredo D. Vitullo

**Affiliations:** 1 Centro de Estudios Biomédicos Básicos, Aplicados y Desarrollo –CEBBAD, Universidad Maimónides, Hidalgo 775, C1405BCK Buenos Aires, Argentina; 2 Consejo Nacional de Investigaciones Científicas y Técnicas, CONICET, Buenos Aires, Argentina

**Keywords:** *FOXO3*, *PTEN*, Cryopreservation, Pre-menarcheal ovary, Post-menarcheal ovary, Ovarian reserve, Extragonadal cancer

## Abstract

**Background:**

*FOXO3/pFOXO3* and *PTEN* expression is known to regulate the dormancy/activation of ovarian primordial follicles. How chemotherapy could influence the expression of *FOXO3* and *PTEN* in pre- and post-menarcheal girls with extra-gonadal cancer remains unexplored.

**Methods:**

Ovarian samples were collected from 27 girls suffering from extra-gonadal cancer. Of these, 8 patients had received chemotherapy before the time of sample collection. Ovarian tissue collected at the time of surgery was fixed in 10% formaldehyde for FOXO3/pFOXO3 and PTEN immunohistochemistry or immunofluorescence, or stored at -80 °C for Western blot, or preserved in RNA later for RT-PCR.

**Results:**

PTEN was detected in a limited number of primordial follicle-enclosed oocytes in approximately fifty percent of the patients, regardless of whether they had received anti-cancer treatment or not. However, there was a significant decrease in PTEN detection in patients who underwent chemotherapy treatment prior to the retrieval of the sample. Both primordial follicle-enclosed oocytes that expressed FOXO3 and those that did not were identified in patients who were treated with chemotherapy and those who were not. FOXO3-positive primordial follicles exhibited either nuclear FOXO3 localization or cytoplasmic pFOXO3 localization. Furthermore, transitional primordial follicles that expressed nuclear FOXO3 and cytoplasmic pFOXO3 were also observed. Primary follicle-enclosed oocytes displayed cytoplasmic pFOXO3 localization, whereas in more advanced stages of folliculogenesis, the expression moved to the somatic stratum. No significant statistical differences were identified in the detection of FOXO3 and pFOXO3 in patients who had or had not received chemotherapy prior to sample collection.

**Conclusion:**

Primordial follicles expressing and not expressing FOXO3 were equally present in both the ovaries of patients who underwent chemotherapy and those who did not. The expression of FOXO3 remained unaltered in response to chemotherapy treatment. Notably, the detection of PTEN was significantly reduced in the treated patients, thereby warranting in-depth investigation, given the limited sample size examined in the present study.

**Supplementary Information:**

The online version contains supplementary material available at 10.1186/s12905-023-02648-x.

## Background

Chemotherapy has been observed to induce damage to the ovary, thereby compromising the patient's fertility. The germinal (follicle reserve) and stromal/vascular compartments are both affected by mechanisms that are not fully understood [1–3]. The extent of the damage that occurs is primarily dependent on the chemotherapy regime utilized as well as the age of the patient at the time of treatment. The ovaries are very sensitive to alkylating agents, which are classified as high risk for gonadal dysfunction, such as cyclophosphamide commonly used in the treatment of a variety of solid tumors and hematological malignancies [4–7]. For this reason, it is important to consider fertility preservation in cancer patients before performing the antitumor treatment. Several options are currently available to preserve fertility in women with cancer: embryo cryopreservation, oocyte cryopreservation, or ovarian tissue cryopreservation. The choice of the most suitable strategy for preserving fertility depends on different parameters including the type and timing of chemotherapy, the type of cancer, the patient’s age, and the partner status [8]. In instances where post-pubertal girls are concerned, the option of oocyte retrieval for cryopreservation may be considered, in addition to ovarian tissue cryopreservation, provided the patient's age and physiological condition are taken into account. However, in the case of pre-pubertal girls, ovarian tissue cryopreservation remains the sole viable option.

The ovarian reserve, comprising of dormant primordial follicles, is of utmost importance for female fertility. Prior to puberty, certain dormant follicles are activated and proceed towards the growing follicular pool where the initial phases of folliculogenesis occur, terminating ultimately in atresia. Following menarche, a hormone-dependent cyclical recruitment of dormant primordial follicles takes place whereby the recruited follicles undergo follicular growth, culminating in the ovulation of a single oocyte during each menstrual cycle while the remaining recruited follicles go through atresia [9–11]. Thus, the maintenance of the ovarian reserve is a complex process that operates in a finely-tuned state of homeostasis, with the goal of preserving a significant proportion of follicles in a dormant state. This serves to enable the successive menstrual cycles that occur throughout a woman's reproductive lifespan, thereby guaranteeing her fertility.

Various genetic factors are implicated in the PI3K/AKT signaling pathway, which plays a crucial role in maintaining the quiescent state or activating the dormant primordial follicle for entry into the growing follicular pool. These genetic factors are expressed either in the germ cell itself or in accompanying granulosa cells [12]. Several growth factors, including FSH (follicle-stimulating hormone), stimulate PI3K to activate a PDK1 (phosphoinositide-dependent protein kinase-1) that will phosphorylate Akt and downstream forkhead transcription factors, FOXOS, that causes follicular activation and growth [13, 14]. In mice, the localization of Foxo3 protein is specifically confined to the oocyte nuclei of primordial follicles, with subsequent translocation to the cytoplasm occurring in primary follicles [15]. This translocation event is linked to the activation and departure from the dormant pool. The regulation of dormancy/activation in primordial follicles of the ovarian reserve in other rodents, such as the rat and deer mouse [16], is also governed by *Foxo3*. However, in contrast to the expression pattern observed in rodents, where *Foxo3* is globally expressed in the nucleus of all dormant primordial follicle-enclosed oocytes and in the cytoplasm in activating primordial follicles, the adult ovary of non-rodent mammals examined thus far does not exhibit detectable *FOXO3* expression in primordial follicles [16]. In humans, two subpopulations of primordial follicles coexist in the postnatal ovary, with one expressing nuclear FOXO3 protein and the other not expressing FOXO3. The prevalence of *FOXO3* expression varies with age, with a low frequency during fetal life that increases during pubertal age and persists into adulthood [17]. It has been postulated that the subset of primordial follicles that express FOXO3 may indeed comprise an especial reservoir that must bide its time for extended periods, potentially spanning years or even decades, before gaining entry into the growing follicular pool [17].

Another factor involved in PI3K/AKT pathway is the *Phosphatase and tension homolog* (*PTEN*) gene. In mice, it has been observed that the deletion of the *Pten* gene stimulates the growth of primordial follicles in neonatal and adult animals [13, 18–20]. This gene encodes for a phosphatase that negatively regulates the PI3K-Akt signaling pathway. Deletion of *Pten* increases the phosphorylation of Akt, inducing its activation. Activated Akt, in turn, phosphorylates and activates *Foxo3*, causing its export from the nucleus to the oocyte´s cytoplasm and the consequent activation of the primordial follicle [14, 19]. In the human ovarian cortical tissue, even short-term *PTEN* inhibition or PI3K activation increases follicle growth and leads to primordial follicle depletion [21]. Activation of the PI3K/PTEN/Akt pathway in oocytes and granulosa cells of primordial follicles in cyclophosphamide-treated mice induces activation of dormant primordial follicles, as demonstrated by increased phosphorylation of Akt, mTOR, and rpS6, and inactivation of the suppressive factor FOXO3. Therefore, cyclophosphamide induces the loss of ovarian reserve because it accelerates the activation of the primordial follicle, which results in a rapid depletion of ovarian follicle reserve [22, 23].

This study aimed to analyze *FOXO3* and *PTEN* expression in ovaries from pre- and post-pubertal girls with extragonadal oncological pathology who had or had not received chemotherapy before surgery to cryopreserve ovarian tissue.

## Materials and methods

### Collection of ovarian samples

A total of 27 small samples of human ovarian tissue were collected and analyzed from pre- and post-pubertal girls entering a program of ovary cryopreservation. All patients had suffered extra-gonadal malignant disease and 8 of them received chemotherapy before surgery (Table 1). In all these 8 patients, samples were taken one month after the end of treatment. At the time of cryopreservation, the patients were aged between 7 and 19 years. Pre- and post-pubertal ovarian tissue samples were obtained by laparoscopic surgery at the “*Hospital de Niños Dr. Ricardo Gutiérrez*”, Buenos Aires, Argentina, including pre-menarcheal patients of 7 (*n* = 1), 9 (*n* = 3), 10 (*n* = 2), 11 (*n* = 1) and 12 (*n* = 1) years old and post-menarcheal patients of 12 (*n* = 2), 13 (*n* = 3), 14 (*n* = 5), 15 (*n* = 4), 16 (*n* = 2), 17 (*n* = 1), 18 (*n* = 3) and 19 (*n* = 1) years old (Table 1). The hospital submitted anonymized medical records with samples indicating the menarcheal condition of the patients. Samples were grouped into patient who received (Group 1; treated-patients 4, 5, 10, 11, 13, 23, 26, 27) or not (Group 2; untreated-patients 1–3, 6–9, 12, 14–22, 24, 25) chemotherapy before surgery (Table 1). Ovarian tissue collected at the time of surgery under sterile conditions was preserved in 10% formaldehyde until embedded in paraffin, serially sectioned at 5 μm thickness, mounted onto cleaned slides, and kept at room temperature until used. When possible (Table S1), two small fresh-tissue fragments were preserved either in an RNAse/DNAse-free sterile cryotube or in 1 ml RNA later (QiaGen, Ambion Inc., Austin, TX, USA) and stored at -80 °C until used. The present study was reviewed and approved by the Institutional Research Ethics Committee, Universidad Maimónides, Buenos Aires, Argentina, and Research Ethics Committee from the collaborating hospital. Samples were utilized only upon obtaining informed consent from patients or, in cases where patients were under the age of sixteen, from their respective parents or legal representatives, in compliance with Argentinean regulations.

**Table 1:** Diagnosis, age, and pre-surgery treatment in oncological patients included in the present study

	**Patient**	**Age (years)**	**Diagnosis**	**Pre-surgery chemotherapy**
**Pre-menarcheal patients**	**1**^**a**^	7	Knee osteosarcoma	No
	**2**	9	Tibia osteosarcoma	No
	**3**	9	Femur osteosarcoma	No
	**4**^**a**^	9	Embryonal rhabdomyosarcoma	Nine cycles of siopMMT95 with ifosfosfamide-vincristine-actinomycin D Six cycles of vincristine-temozolomide-irinotecan after relapse
	**5**^**a**^	10	Hodgkin´s lymphoma	Six cycles of adriamycin-bleomycin-vinblastine-dacarbazine
	**6**	10	Hodgkin´s lymphoma	No
	**7**	11	Tibia sarcoma	No
	**8**	12	Hodgkin´s lymphoma	No
**Post-menarcheal patients**	**9**^**a**^	12	Hodgkin´s lymphoma	No
	**10**^**a**^	12	Acute myeloid leukemia	The first cycle of G.A.T.L.A and the second cycle of ifosfamide and carboplatin etoposide, after relapse
	**11**^**a**^	13	Perineal myosarcoma	Four cycles of ifosfamide-vincristine-actinomycin-epirubicin-etoposide-carboplatin
	**12**^**a**^	13	Shoulder osteosarcoma	No
	**13**^**a**^	13	Inguinal synovial sarcoma	Three cycles of ifosfamide-doxorubicine
	**14**	14	Elbow synovial sarcoma	No
	**15**	14	Knee synovial sarcoma	No
	**16**	14	Hodgkin´s lymphoma	No
	**17**	14	Hodgkin´s lymphoma	No
	**18**^**a**^	14	Rib osteosarcoma	No
	**19**	15	Hodgkin´s lymphoma	No
	**20**	15	pituitary dysgerminoma	No
	**21**	15	Knee sarcoma	No
	**22**^**a**^	15	Hodgkin´s lymphoma	No
	**23**^**a**^	16	Hodgkin´s lymphoma	OPPA scheme. Two cycles of meprednisone-vincristine-doxorubicin-procarbazine. COOP scheme. Two cycles of prednisone-vincristine-procarbazine-cyclophosphamide-mesna
	**24**	18	Hodgkin´s lymphoma	No
	**25**^**a**^	18	Hodgkin´s lymphoma	No
	**26**^**a**^	18	Ganglioneuroblastoma	Cycles 1, 2, 4, and 6 of cyclophosphamide-doxorubicin-vincristine. Cycles 3 and 5 of etoposide-cisplatin. Six cycles of 13-cis-retinoic and 3 cycles of mesnatopothecan. Radiotherapy of 3000 cGy
	**27**^**a**^	19	Myelodysplasic syndrome	cytarabine-idarubicine-etoposide

^a^Indicates patients from which fresh tissue was obtained for western blot and RNA analysis

### Immunohistochemistry

Immunohistochemistry was performed according to Albamonte et al. [24] with some modifications. Briefly, mounted paraffin sections were dewaxed in xylene, rehydrated in graded alcohols, and washed in distilled water. Endogenous peroxidase activity was inhibited with 0.5% H_2_O_2_/methanol (v/v) for 20 min at room temperature. The sections were treated for one hour with 15% normal horse serum or normal rabbit serum in phosphate-buffered saline (PBS) followed by overnight incubation at room temperature with primary antibodies that were diluted 1:100. The primary antibodies used were mouse monoclonal anti-FKHRL1 (FOXO3) (sc-48348), goat polyclonal anti-p-FKHRL1 (pFOXO3) (sc-12357), and mouse polyclonal anti-PTEN (sc-7974), all from Santa Cruz Biotechnology, Dallas, TX, USA. After the incubation period, the slides were rinsed three times in PBS and incubated for one hour with the appropriate 1:200-diluted biotinylated secondary antibody (Vector Labs, Peterborough, UK) at room temperature. The sections were washed again in PBS and then incubated for 30 min with 1:100 diluted streptavidin-peroxidase complexes (ABC kit, Vector Labs, UK). Following this step, the sections were washed twice with PBS and the peroxidase activity was detected with 0.05% 3,3´-diaminobenzidine (w/v) and 0.1% H_2_O_2_ (v/v) in Tris–HCl. Finally, the sections were washed with distilled water and mounted in Canada balsam (Biopack, Buenos Aires, Argentina). Negative controls were processed simultaneously by omitting the primary antibody and/or preincubating the primary antibody with the specific commercial synthetic peptide.

Sections were examined in an Olympus BX40 microscope. To determine the prevalence of primordial follicles expressing or not the antibody used and their cellular localization, the entire processed sections were screened and all primordial follicles counted.

### Double immunofluorescence

Mounted paraffin sections were dewaxed in xylene, rehydrated in graded alcohols, and washed in distilled water. Sections were then blocked for 1 h with bovine serum albumin 15% + bovine fetal serum 10% (w/v) in PBS + tween 0,1% (v/v) and incubated overnight at room temperature with the 1:100 diluted primary antibody mouse polyclonal anti-PTEN (sc-7974). The next day, sections were then washed twice with PBS and incubated for 1 h with secondary antibody anti-mouse Alexa Fluor 488 (1/100, Life Technology). Sections were then washed twice with PBS and incubated overnight at room temperature with the 1:100 diluted primary antibody goat polyclonal anti-p-FKHRL1 (pFOXO3) (sc-12357). The next day, sections were washed twice with PBS and incubated for 1 h with secondary antibody anti-goat Alexa Fluor 555 (1/100, Life Technology). Finally, sections were washed with distilled water and mounted in Vectashield Mounting Medium with DAPI (Vector Laboratories). Negative controls were processed simultaneously by omitting the primary antibody and/or preincubating the primary antibody with the specific commercial synthetic peptide. Sections were examined in a confocal Eclipse Ti microscope (Nikon Instruments Inc. NY, USA).

### Western blot analysis of PTEN

This procedure was performed according to Albamonte et al. [24] with some modifications. Ovarian fragments preserved at -80°C were homogenized in ice-cold lysis buffer containing a protease inhibitor cocktail [0.5 mM phenylmethylsulfonyl fluoride (PMSF); 10 mM leupeptin; 10 mM pepstatin; 10 mM aprotinin], and centrifuged at 1.200* g* at 4°C for 10 min. The supernatant was collected and proteins were quantified using the Bradford Protein Assay (Bio-Rad Laboratories, Inc., Hercules, CA, USA). Total proteins (20 µg) from tissue extracts were separated by one-dimensional SDS-PAGE 15% and then transferred onto polyvinylidene fluoride (PVDF) membrane (Amersham Hybond-P, GE Healthcare). The membrane was then blocked for 1 h in PBS + 0.1% Tween20 with 5% nonfat dry milk. After that, it was incubated for 1 h at room temperature with the mouse polyclonal anti-PTEN diluted 1/100 (A2B1: sc-7974 Santa Cruz Biotechnology. Dallas, TX, USA). After washing, the membrane was incubated with a goat anti-mouse IgG horseradish peroxidase-conjugated secondary antibody (Bio-Rad, 1:5000). The immunoreactive product was visualized using the enhanced chemiluminescence system ECL plus GE (Amersham, Fairfield, Connecticut, USA) and Image Quant 350. To confirm equal loading, each membrane was analyzed for β-actin protein expression demonstrating that the band intensities did not show significant changes between the samples analyzed. Briefly, the membrane was incubated with mouse monoclonal anti-β-actin (Sigma, Saint Louis, Missouri, USA) diluted 1/1000. After washing, the membrane was incubated with a goat anti-mouse IgG horseradish peroxidase-conjugated secondary antibody (Bio-Rad, 1:5000). Stained protein molecular weight markers were used as standards (Fermentas, Vilnius, Lithuania). Densitometry was performed on Scion Image for Windows software (Scion Corporation, Frederick, MD, USA) and PTEN expression was normalized to β-actin. The human uterine fibroblast (HUF) cell line was utilized as a positive control for PTEN expression [25].

### RNA isolation and real time-PCR

The present protocol was performed according to Albamonte et al. 2020 [24] with some modifications. Samples recovered in RNA later in the surgery room were maintained in that solution for 48 h and then stored a -80 °C until used. RNA was extracted from the ovary using Trizol (Invitrogen, Waltham, MA, USA) in accordance with the manufacturer's instructions. Subsequently, DNAse I (Invitrogen, Waltham, MA, USA) was applied to 3 μg of total RNA, followed by reverse transcription using a 20 μl-reaction containing M-MLV reverse transcriptase (200 U/μl, Promega, Madison, WI, USA) and random hexamers primers (Biodynamics, Buenos Aires, Argentina). Finally, quantitative polymerase chain reaction (PCR) was conducted utilizing reverse-transcribed cDNA, specific forward (F) and reverse (R) primers, and SYBR Green PCR Master Mix in a Stratagene MPX500 cycler (Stratagene, La Jolla, CA, USA). Specific primers used were: *PTEN*; F 5´- CCAATGTTCAGTGGCGGAACT-3´, R 5´- GAACTTGTCTTCCCGTCGTGT-3´; F*OXO3*: F 5´-TCTACGAGTGGATGGTGCGTT-3´, R 5´- CGACTATGCAGTGACAGGTTGT-3´; *ACTIN*: F 5´- CTTCCCCTCCATCGTGGG-3´, R 5´- GTGGTACGGCCAGAGGCG-3´*)*. Primers were used at a concentration of 0.3 μM in each reaction. The cycling conditions were as follows: step 1, 10 min at 95 °C; step 2, 15 s at 95 °C; step 3, 30 s at 60 °C; step 4, 30 s at 72 °C, repeating steps 2 to 4 forty-five times. Data from the reaction were collected and analyzed by the complementary computer software (MxPro3005P v4.10 Build 389, Schema 85, Stratagene, La Jolla, CA, USA). Melting curves were run to confirm the specificity of the signal. Relative quantitation of gene expression was performed using standard curves and normalized to β-actin in each sample. To evaluate quantitative differences in the cDNA target among samples, we employed the Pfaffl mathematical model. The expression ratio was determined for each sample by calculating (Etarget)ΔCt(target)/(EGβACTIN)ΔCt(βactin), where E is the efficiency of the primer set and CT is threshold cycle with ΔCt = Ct (normalization cDNA)—Ct (experimental cDNA). The amplification efficiency of each primer set was calculated from the slope of a standard amplification curve of log (ng cDNA) per reaction vs. Ct value (E = 10-(1/slope)). Efficiencies of 2 ± 0.1 were considered optimal.

### Statistical analysis

Analysis of the data entailed calculating the mean and standard error (SEM), with one-way analysis of variance being conducted using InfoStat Software (Version 2012, Grupo InfoStat, Universidad Nacional de Córdoba, Córdoba, Argentina). A log10 transformation of the data was performed, and Tukey's test was conducted when comparing differences between more than two groups. A *p*-value of less than 0.05 was considered statistically significant.

## Results

### PTEN protein detection in pre- and post-pubertal human ovaries

**Fig. 1:**
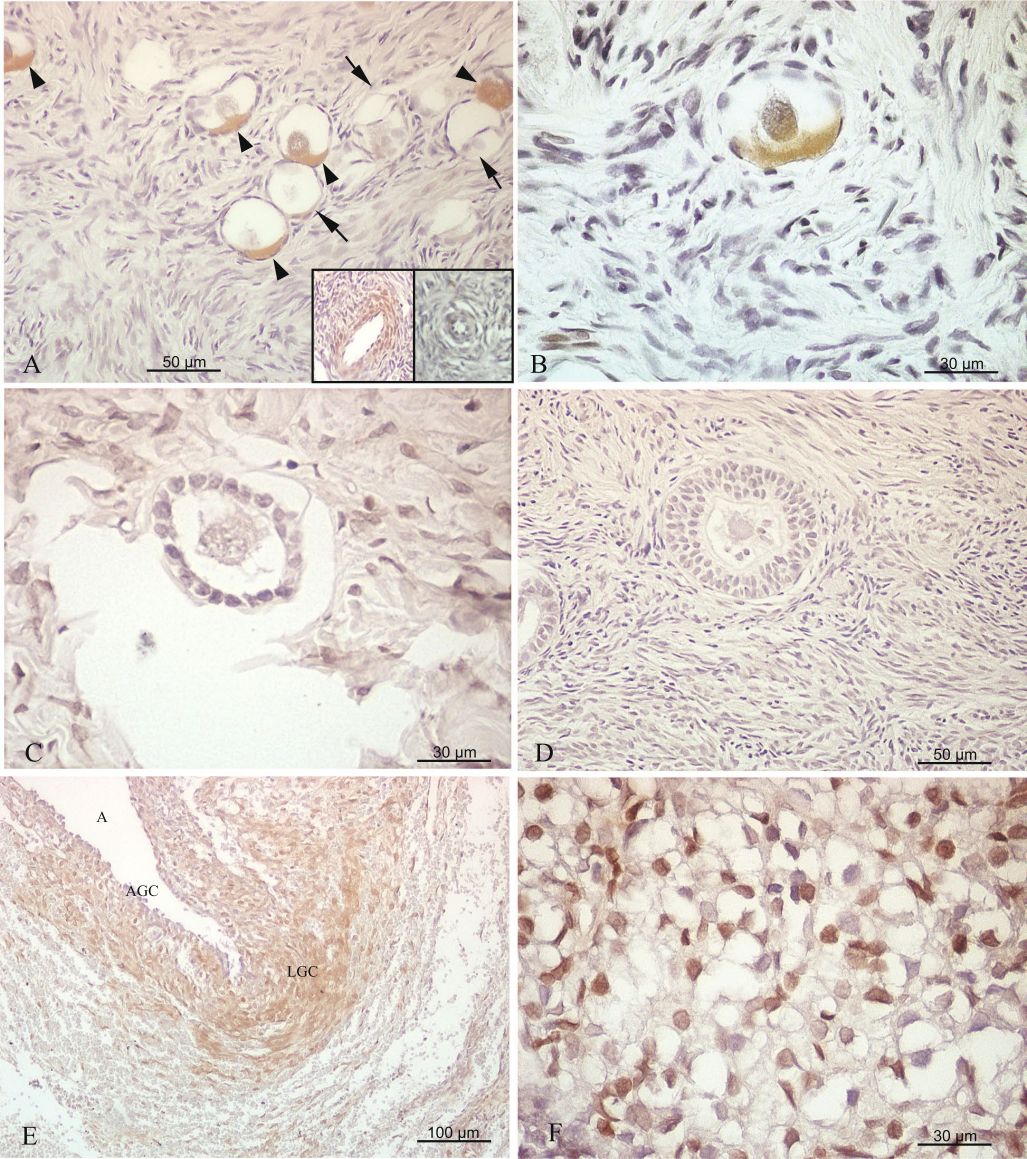
Immunodetection of PTEN in the pre- and post-pubertal human ovary with extragonadal oncopathology. **A** PTEN-immunoreactive (arrowheads) and PTEN-negative (arrows) primordial follicles in a 9-year-old patient that did not receive chemotherapy treatment before sample collection. **B** Detail of a primordial follicle with cytoplasmic immunoreactivity for PTEN from a 9-year-old patient who received chemotherapy before sample collection. **C** Primary follicle negative for PTEN (untreated patient). **D** Secondary follicle negative for PTEN (untreated patient). **E** Atretic antral follicle with granulosa cells (AGC) and luteal granulosa cells (LGC) positive for (untreated patient); A, antrum. **F** Detail of luteal body positive for PTEN (treated patient). Insets in (**A**) show PTEN immunoreactive blood vessels (right inset) from the same section, used as internal positive antibody reactivity control, and negative PTEN blood vessel when primary antibody was preabsorbed with the specific commercial synthetic peptide (left inset)

**Fig. 2:**
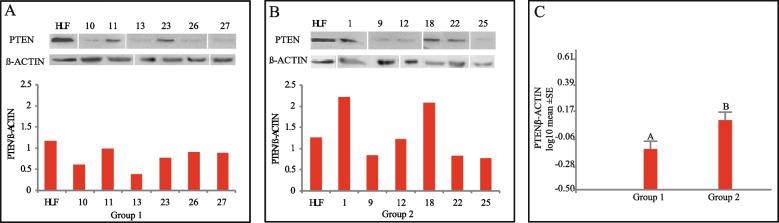
Western blot quantification of PTEN protein in pre- and post-pubertal human ovaries. **A** Patients that received chemotherapy before cryopreservation. **B** Patients that did not receive chemotherapy before cryopreservation. **C** PTEN expression level comparison between the chemotherapy-treated and untreated group. The bars represent the values expressed as mean ± ES. Different letters indicate significant differences between groups (Tukey, α: 0.05, *p* < α). Group 1, treated-patients; Group 2, untreated-patients. Numbers over lanes and under bars in A and B indicate the patient number (see Table S1). HUF, Human Uterine Fibroblast cell line. Original, full-length gels from where clipped bands were extracted are shown in Fig. 1S

The majority of primordial follicles did not show PTEN immunoreactivity, regardless of whether or not patients received chemotherapy. However, half of the patients from both groups showed, in general, a low percentage of primordial follicles positive for cytoplasmic PTEN (Fig. 1A, B; Table 2). No PTEN signal was detectable in primary and secondary follicles (Fig. 1C-D) whereas antral, atretic antral follicles, and corpora lutea showed only PTEN-positive somatic cells (Fig. 1E–F). Overall quantification of PTEN-expressing primordial follicles was significantly lower (3.62%) in Group 1 (treated-patients) than in Group 2 (11.12%; untreated-patients) (Tukey test, α:0.05; *p* < 0.0001, see Table 2). PTEN detection by western blot was low in all patients from both groups (Fig. 2A-B). However, PTEN expression was significantly lower in Group 1 (Tukey test, α:0.05; *p* < 0.0001) (Fig. 2C).

**Table 2:** Counting of PTEN-positive and PTEN-negative primordial follicles in patients that received (Group 1) and not received (Group 2) chemotherapy and/or radiotherapy before surgery for cryopreservation

	**Patient**	**Age (years)**	**Total Follicle count**	**Primordial follicle**	
				**PTEN**^**−**^	**PTEN**^**+**^
**Group 1**	**4**	9	58	51	7
	**5**	10	29	29	0
	**10**	12	70	70	0
	**11**	13	31	30	1
	**13**	13	74	72	2
	**23**	16	23	22	1
	**26**	18	9	9	0
	**27**	19	10	10	0
	**Total Count**		**304**	**293**	**11**
	**Total Count (%)**		**100,00**	**96,38**	**3,62**^**#**^
**Group 2**	**1**	7	25	24	1
	**2**	9	40	40	0
	**3**	9	33	11	22
	**6**	10	21	21	0
	**7**	11	104	59	45
	**8**	12	41	41	0
	**9**	12	8	8	0
	**12**	13	13	13	0
	**14**	14	31	31	0
	**15**	14	61	58	3
	**16**	14	272	253	19
	**17**	14	30	18	12
	**18**	14	15	15	0
	**19**	15	48	46	2
	**20**	15	179	177	2
	**21**	15	49	47	2
	**22**	15	167	167	0
	**24**	18	35	12	23
	**25**	18	10	9	1
	**Total Count**		**1.182**	**1.050**	**132**
	**Total Count (%)**		**100,00**	**88,83**	**11,17**^**#**^

^#^Percentage PTEN^+^ values for groups 1 and 2 exhibited statistically significant differences; Tukey test, α:0.05; *p* < 0.0001

### FOXO3 and pFOXO3 protein detection in pre- and post-pubertal human ovaries

**Fig. 3:**
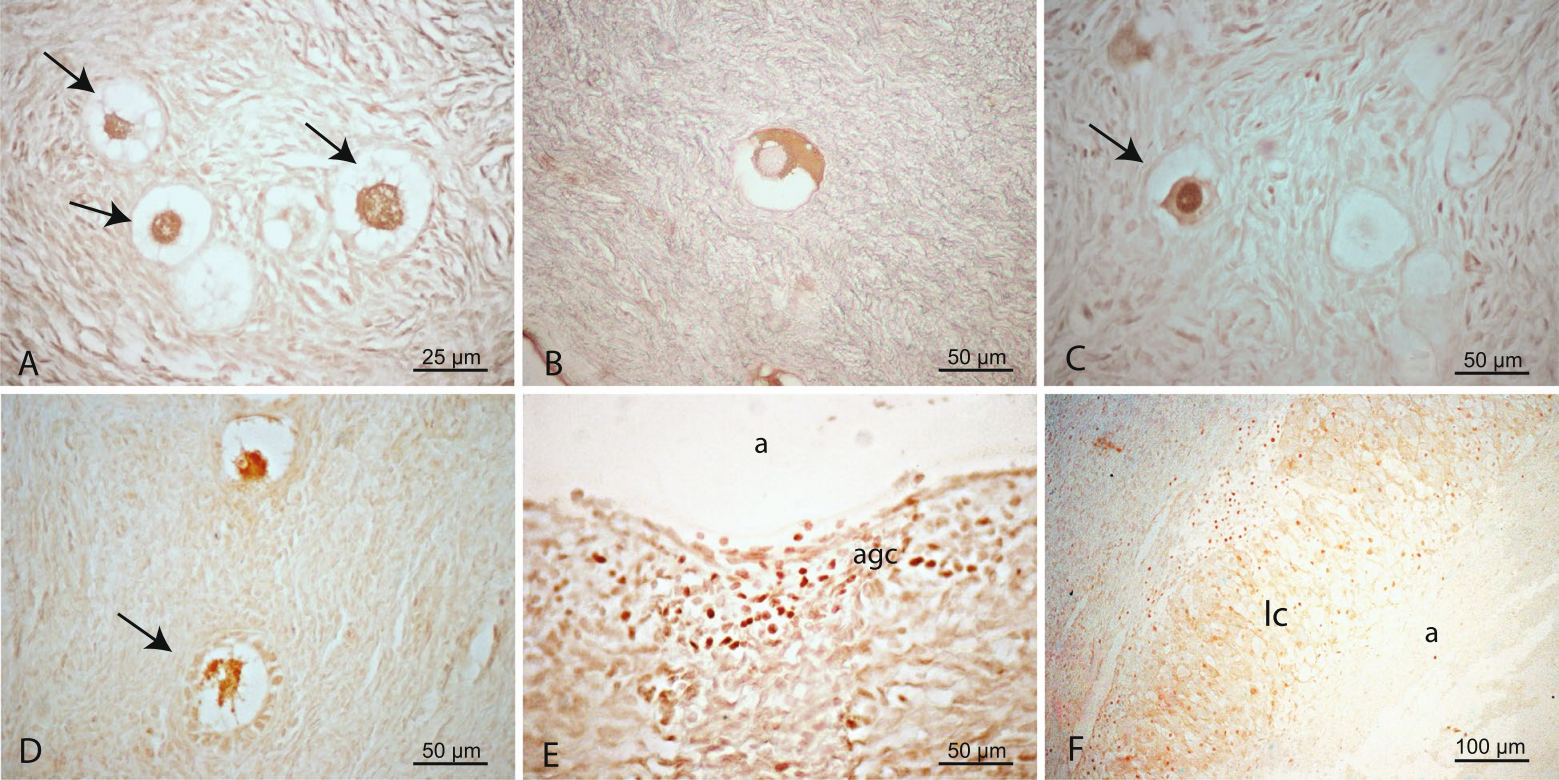
Immunohistochemical detection of FOXO3 and pFOXO3 in pre- and post-pubertal human ovaries with extragonadal oncopathology. **A** Primordial follicles (arrows) with nuclear localization of FOXO3. **B** Primordial follicle with cytoplasmic localization of pFOXO3. **C** Transitional follicle (arrow) displaying nuclear FOXO3 and cytoplasmic pFOXO3 expression. **D** Primary follicle (arrow) positive for FOXO3. **E** Detail of an atretic antral follicle positive for FOXO3 in granulosa cells (agc); a, antrum. **F** Detail of a luteal body positive for FOXO3; lc, luteal cells; a, antrum

**Fig. 4:**
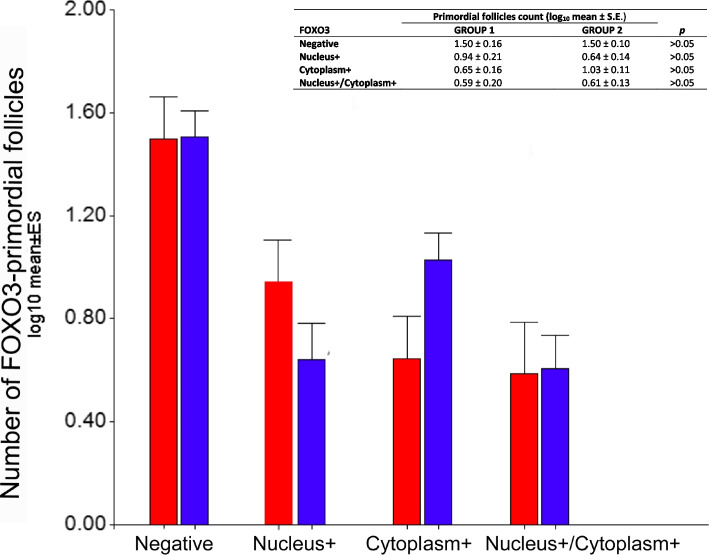
Quantification of primordial follicles positive for FOXO3 in pre- and post-pubertal human ovaries. Red bars, mean value for chemotherapy-treated patients; blue bars, untreated patients before cryopreservation. Negative, no immunoreactive primordial follicles; N + , primordial follicles with FOXO3 nuclear expression; C + , primordial follicles with cytoplasmic pFOXO3 expression; N + /C + , transitional primordial follicles displaying both nuclear FOXO3 and cytoplasmic pFOXO3 immunoreactivity

FOXO3 protein showed a variable pattern of expression in the pool of primordial follicles in both treated and untreated patients. Some primordial follicles were negative while others showed nuclear expression of FOXO3, cytoplasmic expression of pFOXO3, or both (transitional follicles) (Fig. 3A-C). Oocytes in primary follicles were positive for cytoplasmic FOXO3. On the other hand, secondary, antral, atretic follicles, and corpora lutea showed FOXO3 expression in somatic cells, in both groups (Fig. 3D-F). The quantification of primordial follicles displaying the different cellular localization of FOXO3/pFOXO3 in both groups (Table 3) did not show significant statistical differences (Fig. 4). Cytoplasmic pFOXO3 was found in oocytes of primary follicles and was not detected in secondary, antral, and atretic follicles, and luteum bodies.

**Table 3:** Counting of FOXO3-positive and FOXO3-negative primordial follicles in patients that Received (Group 1) or not received (Group 2) chemotherapy and/or radiotherapy before surgery for cryopreservation

	**Patient**	**Age (years)**	**Total Follicle count**	**Primordial follicle**			
				**-**	**N + **	**C + **	**N + /C + **
**Group 1**	**4**	9	51	46	0	5	0
	**5**	10	122	90	28	2	2
	**10**	12	94	38	43	4	9
	**11**	13	110	67	10	26	7
	**13**	13	82	11	41	5	25
	**23**	16	45	38	4	1	2
	**26**	18	31	25	1	4	1
	**27**	19	20	9	2	7	2
	**Total Count**		**555**	**324**	**129**	**54**	**48**
	**Total Count (%)**		**100,00**	**58,38**	**23,24**	**9,73**	**8,65**
**Group 2**	**1**	7	41	23	9	9	0
	**2**	9	71	51	3	7	10
	**3**	9	86	42	0	21	23
	**6**	10	30	21	5	3	1
	**7**	11	223	133	0	48	42
	**8**	12	105	75	3	22	5
	**9**	12	15	10	0	4	1
	**12**	13	58	20	4	32	2
	**14**	14	38	30	2	1	5
	**15**	14	67	40	2	21	4
	**16**	14	269	246	5	11	7
	**17**	14	37	19	1	15	2
	**18**	14	19	15	0	3	1
	**19**	15	98	23	20	55	0
	**20**	15	123	117	2	3	1
	**21**	15	97	66	3	5	23
	**22**	15	385	229	126	30	0
	**24**	18	79	29	10	28	12
	**25**	18	18	8	1	8	1
	**Total Count**		**1.859**	**1.197**	**196**	**326**	**140**
	**Total Count (%)**		**100,00**	**64,39**	**10,54**	**17,54**	**7,53**

### pFOXO3 and PTEN double immunofluorescence labelling 

**Fig. 5:**
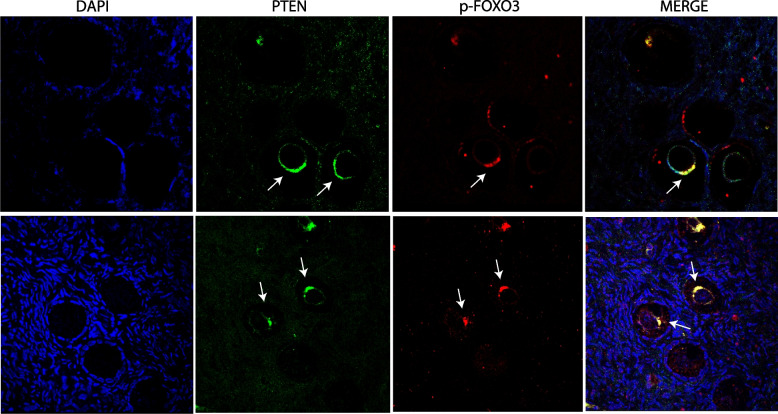
Double immunolabeling for pFOXO3 and PTEN in pre- and post-pubertal human ovary with extragonadal oncopathology. Arrows indicate primordial follicles positive for PTEN, pFOXO3, or both (merge)

Protein co-localization showed, in both groups, positive or negative primordial follicles for both pFOXO and PTEN as well as positive primordial follicles for both proteins. In follicles showing both proteins, they co-localized in the form of cytoplasmic aggregates around the oocyte nucleus (Fig. 5).

### mRNA *FOXO3* and *PTEN* expression in pre- and post-pubertal human ovaries

**Fig. 6:**
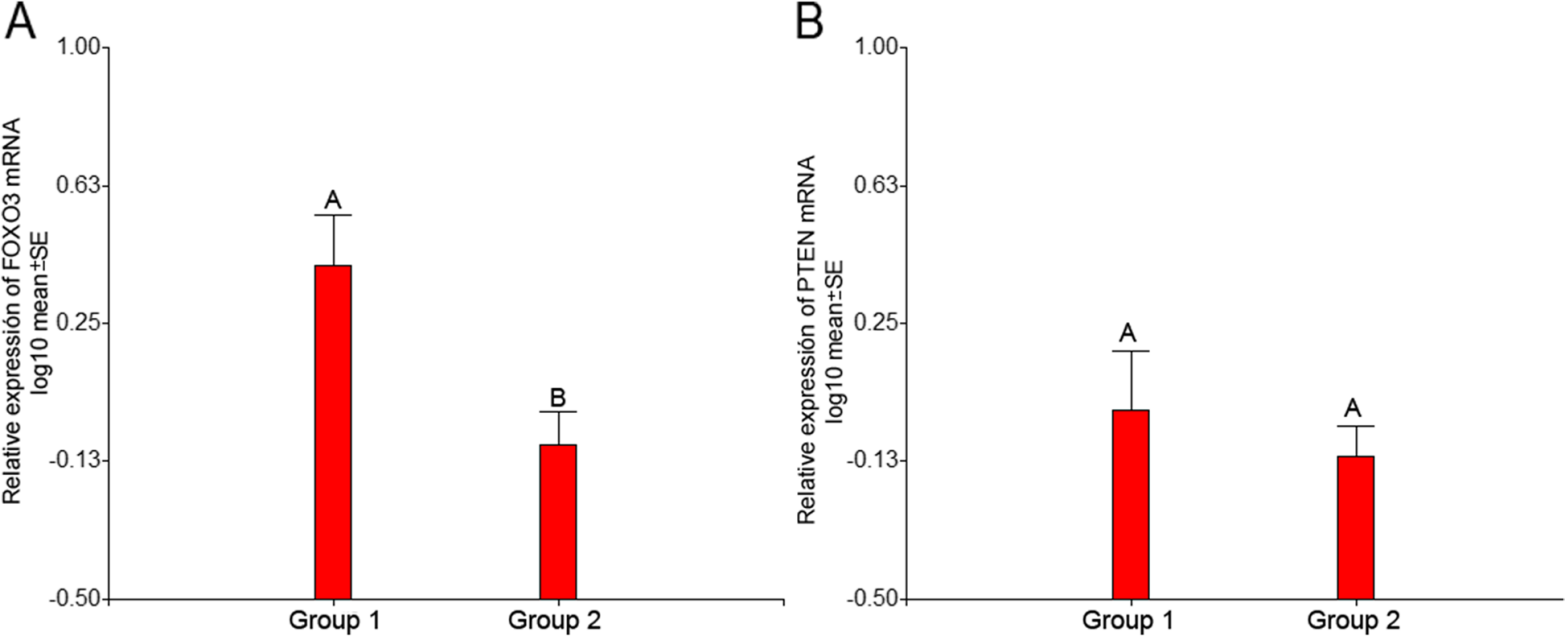
mRNA quantification for *FOXO3* and *PTEN*. **A** Mean value of *FOXO3* transcript amount was significantly different in chemotherapy-treated-patients (Group 1) (mean ± s.e., 0.41 ± 0.13) than in untreated-patients (Group 2) (mean ± s.e., -0.08 ± 0.09). **B** No differences were detected in *PTEN*-mRNA mean values (0.01 ± 0.16 in Group 1 vs. -0.11 ± 0.08 in Group 2). Different letters over the bars indicate significant statistical differences

The quantification of *FOXO3* and *PTEN* mRNAs is shown in Fig. 6. Mean values of *FOXO3* transcripts (Fig. 6A) were significant different in treated- and untreated-patients while no differences were seen for *PTEN* (Fig. 6B).

## Discussion

In mice, the regulation of activation of dormant primordial follicles depends on the translocation of Foxo3 protein from the nucleus to the cytoplasm of the oocyte [13]. The nuclear localization of Foxo3 protein has a potent effect in maintaining the dormancy of the primordial follicle. This effect is evident in *Foxo3*^*−/−*^ mice in which primordial follicles begin to grow uncontrollably until the follicular reserve is depleted [15]. Similarly, other muroid rodents, v.g. rats and deer mice are believed to regulate dormancy/activation through *Foxo3* expression [16]. However, in non-rodent mammals so far analyzed no FOXO3/pFOXO3 was detectable in the nucleus/cytoplasm of quiescent/activating primordial follicles [16]. In the human ovary, we have previously reported that nuclear *FOXO3*-expressing and non-*FOXO3*-expressing primordial follicle subpopulations coexist from fetal life to maturity and that prevalence of *FOXO3*-expressing primordial follicles increases from birth with age [17]. In agreement with this, the results presented here confirm the co-existence of these two subpopulations in the resting primordial follicle pool. Moreover, follicles with nuclear localization of FOXO3, cytoplasmic localization of pFOXO3, and also the so-called transition follicles with nuclear and cytoplasmic localization were detected in both chemo/radiotherapy treated and untreated groups (cf. Table 3). pFOXO3 is also detectable in the cytoplasm of primary follicle-enclosed oocytes. In more advanced stages of folliculogenesis, FOXO3/pFOXO3 localizes in the somatic stratum. Although no statistically significant differences were detected in the number of *FOXO3*-expressing primordial follicles between both groups (see Fig. 4), samples from anti-cancer treated patients showed a tendency to have more primordial follicles positive for nuclear FOXO3 and less cytoplasmic pFOXO3. This observation deserves further investigation to elucidate if non-*FOXO3*-expressing primordial follicles are more susceptible to damage caused by anticancer drugs or, in other words, if the presence of nuclear FOXO3 confers some protection to primordial follicles.

In a previous report, we failed to consistently detect PTEN in primordial follicles from pre- and post-pubertal ovaries, except in one case, probably due to the low number of patients and especially to the scarce number of primordial follicles in processed samples [17]. In the present study, we observed *PTEN*-expressing primordial follicles in approximately half of the patients in each group. However, the group that had received chemotherapy before surgery showed significantly lower expression of *PTEN*. The PI3K/PTEN/Akt signaling pathway is responsible for the control of follicle quiescence and activation. PTEN regulates the balance of this pathway and is essential for maintaining a state of dormancy [26]. Chemotherapy agent cyclophosphamide triggers upregulation of the PI3K pathway, initiating a wave of follicle recruitment and growth and, ultimately, burnout of the ovarian follicle reserve [22]. Therefore, the decrease in PTEN observed in the group of patients that received chemotherapy before surgery could cause phosphorylation of AKT by PIP3. Phosphorylated and active AKT, would in turn phosphorylate nuclear FOXO3 causing its export to the cytoplasm of the oocyte of the primordial follicle with its consequent activation. Although a decrease in primordial follicles is not observed in Group 1 versus Group 2 patients, the decrease in PTEN in Group 1 could indicate the intrinsic damage suffered in that gonad. Six out of the 8 samples included in Group 1 were previously analyzed by us for the presence of apoptotic markers [27]. The presence of cleaved-caspase 3 in primordial follicles suggested that the process of cell damage is already at play in some follicles that are going to be recruited to the growing pool and most likely they will enter atresia during or before reaching the antral stage. Only antral follicles, and especially fully-grown follicles, as well as atretic follicles, showed TUNEL and cleaved-caspase 3 positive signals in granulosa and theca cells, indicating an active apoptotic process accompanying follicular atresia. This is reinforced by the detection of BAX and the FAS/FAS-L system in the somatic layer of these follicles [27]. PTEN and FOXO3 detection in the somatic layer of antral, atretic follicles, and luteum body can favor the atresia by apoptosis [28–33] since FOXO3 activates apoptosis through upregulation of BH3-only proteins or extrinsic apoptotic factors such as FASL and TRIAL [34]. Nevertheless, as previously reported, these 6 patients from Group 1 showed anti-apoptotic markers such as BCL2 and a high detection of nuclear OCT3/4 protein in the primordial pool. This could be a response to the increased proliferative activity of ovarian stem cells indicating that germ cells are responding to survive in a stressful environment being a good prognosis fact for an eventual future recovery of gametogenic capacity [27].

Therapeutic advances in oncology have improved survival rates, partially shifting the focus from surviving cancer to preserving an optimal quality of life after completing treatments and this includes becoming a biological mother [35–37]. The main technique available to preserve fertility in pediatric patients is ovarian tissue cryopreservation. Despite being considered an experimental technique, currently, more than 130 pregnancies have been reported after autologous ovarian tissue transplantation [38–41]. According to the National Institute of Cancer from Argentina, the pediatric cancer cure rate is greater than 70% (www.argentina.gob.ar/salud/instituto-nacional-del-cancer/institucional/roha); therefore, it is increasingly recommended to provide fertility preservation counseling in girls, adolescents, and young women with cancer before indicating a specific cancer treatment [42].

Understanding genes and regulatory pathways involved in the maintenance of dormancy/activation of primordial follicles, as those in this report, are of great interest for clinical improving the chances of in vitro follicular development after thawing of the cryopreserved ovary, and require further in-depth investigation.

### Supplementary Information

**Additional file 1: Table S1.** Repertory of ovarian samples included in this study.

**Additional file 2. **

## Data Availability

All data generated or analysed during this study are included in this published article [and its supplementary information files].
