# Supplementary material for: FOXO3 and PTEN expression in the ovary of girls with extra-gonadal cancer with or without chemotherapy treatment prior to cryopreservation
Source: BMC Womens Health. 2023 Sep 22;23:509. doi: 10.1186/s12905-023-02648-x (PMC10515424; doi:10.1186/s12905-023-02648-x)
Supplement: Supplementary file 1 — Additional file 1: Table S1. Repertory of ovarian samples included in this study. [file 12905_2023_2648_MOESM1_ESM.docx]

**FOXO3 and PTEN expression in the ovary of girls with extra-gonadal cancer with or without chemotherapy treatment prior to cryopreservation.**

M. Itatí Albamonte^a^, Lara Y. Calabró^a^, Mirta S. Albamonte^a^ & Alfredo D. Vitullo^a,b^

^a^Centro de Estudios Biomédicos Básicos, Aplicados y Desarrollo –CEBBAD, Universidad Maimónides, C1405BCK-Buenos Aires, Argentina.

^b^Consejo Nacional de Investigaciones Científicas y Técnicas, CONICET, Argentina.

Supplementary Data

**Table S1. Repertory of ovarian samples included in this study.**

| **Patient** | **Age (years)** | **Fixed Tissue** | **Fresh Tissue** |
| --- | --- | --- | --- |
| 1 | 7 | YES | YES |
| 2 | 9 | YES | NO |
| 3 | 9 | YES | NO |
| 4 | 9 | YES | NO |
| 5 | 10 | YES | NO |
| 6 | 10 | YES | NO |
| 7 | 11 | YES | NO |
| 8 | 12 | YES | NO |
| 9 | 12 | YES | YES |
| 10 | 12 | YES | YES |
| 11 | 13 | YES | YES |
| 12 | 13 | YES | YES |
| 13 | 13 | YES | YES |
| 14 | 14 | YES | NO |
| 15 | 14 | YES | NO |
| 16 | 14 | YES | NO |
| 17 | 14 | YES | NO |
| 18 | 14 | YES | YES |
| 19 | 15 | YES | NO |
| 20 | 15 | YES | NO |
| 21 | 15 | YES | NO |
| 22 | 15 | YES | YES |
| 23 | 16 | YES | NO |
| 24 | 16 | YES | YES |
| 25 | 17 | YES | NO |
| 26 | 18 | YES | NO |
| 27 | 18 | YES | YES |
| 28 | 18 | YES | YES |
| 29 | 19 | YES | YES |
