# Supplementary material for: FOXO3 and PTEN expression in the ovary of girls with extra-gonadal cancer with or without chemotherapy treatment prior to cryopreservation
Source: BMC Womens Health. 2023 Sep 22;23:509. doi: 10.1186/s12905-023-02648-x (PMC10515424; doi:10.1186/s12905-023-02648-x)
Supplement: Supplementary file 2 — Additional file 2. [file 12905_2023_2648_MOESM2_ESM.docx]

**FOXO3 and PTEN expression in the ovary of girls with extra-gonadal cancer with or without chemotherapy treatment prior to cryopreservation.**

M. Itatí Albamonte, Lara Y. Calabró, Mirta S. Albamonte & Alfredo D. Vitullo

# Spplementary Figure 2

Patient 13 27 26 9 12 22 23 1 25 10 26 18 11


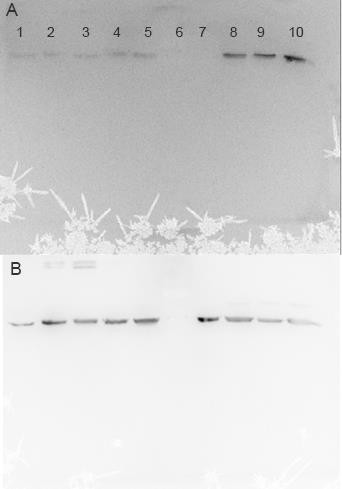

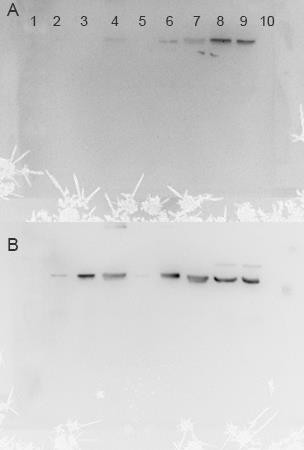


Patient 25 29 23 27 10 9 11 18 1 12 23 13 22 26

#
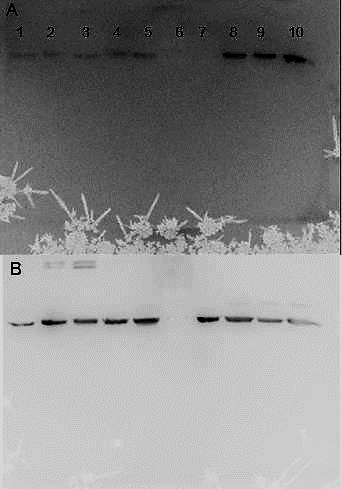

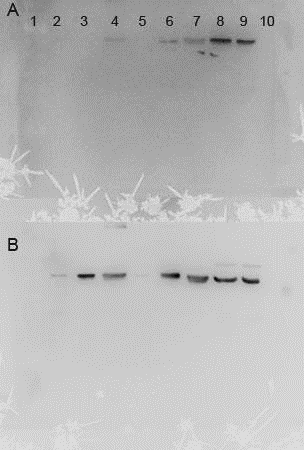


Original, full-length gels from which bands (delineated in red) shown in Fig. 2A, B in the main text were clipped. In all cases upper gel (A) corresponds to PTEN and lower gels (B) are β-actin. Number over the gels indicate the number of patient as shown in the main text (Cf. Fig. 2A, B); not red-delineated bands correspond to other experiments not included in this study.
